# Phosphorylation of HORMA-domain protein HTP-3 at Serine 285 is dispensable for crossover formation

**DOI:** 10.1093/g3journal/jkac079

**Published:** 2022-04-07

**Authors:** Debabrata Das, Shalini Trivedi, Jitka Blazícková, Swathi Arur, Nicola Silva

**Affiliations:** 1 Department of Genetics, University of Texas MD Anderson Cancer Center, Houston, TX 77030, USA; 2 Department of Biology, Faculty of Medicine, Masaryk University, 62500 Brno, Czech Republic

**Keywords:** *Caenorhabditis elegans* meiosis, HORMA-domain proteins, HTP-3

## Abstract

Generation of functional gametes is accomplished through a multilayered and finely orchestrated succession of events during meiotic progression. In the *Caenorhabditis elegans* germline, the HORMA-domain-containing protein HTP-3 plays pivotal roles for the establishment of chromosome axes and the efficient induction of programmed DNA double-strand breaks, both of which are crucial for crossover formation. Double-strand breaks allow for accurate chromosome segregation during the first meiotic division and therefore are an essential requirement for the production of healthy gametes. Phosphorylation-dependent regulation of HORMAD protein plays important roles in controlling meiotic chromosome behavior. Here, we document a phospho-site in HTP-3 at Serine 285 that is constitutively phosphorylated during meiotic prophase I. pHTP-3^S285^ localization overlaps with panHTP-3 except in nuclei undergoing physiological apoptosis, in which pHTP-3 is absent. Surprisingly, we observed that phosphorylation of HTP-3 at S285 is independent of the canonical kinases that control meiotic progression in nematodes. During meiosis, the *htp-3*(*S285A*) mutant displays accelerated RAD-51 turnover, but no other meiotic abnormalities. Altogether, these data indicate that the Ser285 phosphorylation is independent of canonical meiotic protein kinases and does not regulate HTP-3-dependent meiotic processes. We propose a model wherein phosphorylation of HTP-3 occurs through noncanonical or redundant meiotic kinases and/or is likely redundant with additional phospho-sites for function in vivo.

## Introduction

Sexual reproduction relies on the formation of haploid gametes through meiosis, a specialized cell division mechanism that ensures equal distribution of the genetic material in the daughter cells (Zickler and Kleckner 1999, 2015). Faithful chromosome segregation depends on the recognition of the homologous chromosomes (pairing), stabilization of their association through the synaptonemal complex (SC; synapsis), and establishment of chiasmata (recombination; Zickler and Kleckner 1999, 2015). The latter arises from crossover (CO)-dependent repair of programmed double-strand breaks (DSBs), which are generated during meiotic prophase I by the topoisomerase-like enzyme Spo11 (Keeney *et al.* 1997). The SC is a proteinaceous tripartite structure composed of lateral and central elements that assembles in a “zipper”-like fashion to keep the homologous chromosomes tightly juxtaposed, thus allowing for the physical exchange of DNA molecules during homology-mediated DSB repair.

A family of HORMA-domain-containing proteins, composed of HTP-1/2, HTP-3, and HIM-3 in *Caenorhabditis* *elegans*, localizes along the axial elements of the SC and exerts essential functions for axes morphogenesis and the licensing of the SC loading between the homologs (Couteau *et al.* 2004; Martinez-Perez and Villeneuve 2005; Goodyer *et al.* 2008; Severson *et al.* 2009). HTP-3 forms the base of the scaffold, and lack of HTP-3 prevents (1) SC polymerization by abolishing proper formation of chromosome axes and (2) a dramatic reduction of recombination intermediates, indicating an important role for this protein in the efficient induction of meiotic DSBs (Goodyer *et al.* 2008). Recent evidence has shown that HTP-3 can be directly regulated by kinases during meiotic progression. For example, Das *et al.* (2020) observed that ERK/MPK-1 phosphorylates HTP-3 in vitro, however, the actual phosphorylation site remains to be determined.

We have previously shown that *parg-1*, the nematode ortholog of mammalian poly(ADP-ribose) glycohydrolase *PARG*, is an important factor required to coordinate DSB induction and repair and identified physical interactors of PARG-1 in worms (Janisiw *et al.* 2020). In a mass spectrometry analysis performed on PARG-1*::*GFP pull downs, we identified a putative phosphorylated form of HTP-3 at Serine 285, which we further investigated. A phospho-specific HTP-3^S285^ antibody detected phosphorylation of HTP-3 throughout meiotic prophase I, confirming that this site is phosphorylated in vivo. Phosphorylated-HTP-3^S285^ localization overlaps with that of total HTP-3 and is independent of meiotic DSBs, synapsis, or COs. Individual removal of canonical meiotic kinases did not alter HTP-3^S285^ phosphorylation pattern, suggesting that HTP-3 may be phosphorylated at multiple sites and these phospho-sites may function redundantly. Alternatively, S285 may be phosphorylated by multiple kinases in a redundant manner. Preventing HTP-3^S285^ phosphorylation in vivo does not impair axes morphogenesis and recombination, indicating that this site is not essential to successfully achieve chiasmata formation. Altogether, our data show that phosphorylation of HTP-3 at Ser285 likely functions in a redundant manner with other phospho-residues that are yet to be identified.

## Materials and methods

### *Caenorhabditis elegans* genetics and viability assays

The Bristol N2 *C. elegans* strain (Brenner 1974) was used as the wild-type control. The *htp-3(S285A)* was generated by CRISPR/Cas9 genome editing by SUNY Biotech. Silent mutations encoding for an *HhaI* restriction site were included for screening purposes. The strains generated by CRISPR/Cas9 were outcrossed to wild-type N2 worms at least twice before use. All strains were maintained at 20°C under standard conditions for all experiments unless otherwise indicated. Viability and male progeny assessment were performed on single animals plated as L4 and then moved onto fresh NG plates every 24 h for 3 days. Dead embryos/total embryos were scored 24 h after the mother had been moved and the presence of males was evaluated 3 days later. Strains used for this study were:

CA1199: *unc-119(ed3)* III*; ieSi38 [sun-1p::TIR1::mRuby::sun-1 3'UTR + Cbr-unc-119(+)]* IV.

TY5038: *htp-3(tm3655)* I*/hT2 [bli-4(e937) let-?(q782) qIs48] (*I,III*).*

PHX3069*: htp-3[syb3069(S285A)]* I.

AV590: *cosa-1(tm3298)* III*/qC1 [dpy-19(e1259) glp-1(q339)]* III.

AV106: *spo-11(ok79)* IV*/nT1 [unc-?(n754) let-?] (*IV;V*).*

AV276: *syp-2(ok307)* V*/nT1 [unc-?(n754) let-?(m435)] (*IV;V*).*

RB1583: *plk-2(ok1936)* I.

ATG330: *chk-2(fq41[chk-2::AID])* V*; ieSi38* IV.

YKM295: *GFP::cosa-1* II*; AID::cdk-1* III*; ieSi38* IV.

YKM110: *cdk-2::AID::3xFLAG* I*; GFP::cosa-1* II*; ieSi38* IV.

YKM388: *cdk-2::AID::3xFLAG* I*; AID::cdk-1* III*; GFP::cosa-1* II*, ieSi38* IV.

NSV363: *atm-1(gk186)* I*; atl-1(tm853)* V*/nT1 [unc-?(n754) let-? qIs50] (*IV;V*).*

RB1562: him-5(ok1896) V.

VC172: cep-1(gk138) I.

NSV228: brc-1(ddr41) III.

### RNAi experiments and auxin treatment

RNAi experiments were conducted by feeding L4 staged animals on HT115(DE3) bacteria expressing dsRNA of the relevant target gene (*chk-1*) for 48 h until dissection.

NGM plates containing 1-mM 5-Hydroxyindole-3-acetic acid (Sigma) dissolved in absolute ethanol (+auxin) were used for auxin-induced degradation of *cdk-1*, *cdk-2*, and *chk-2.* The control plates (-auxin) were poured in the same way with the addition of an identical volume of ethanol without auxin. Given that presence of auxin inhibits bacterial growth, a saturated OP50 culture was concentrated 5× before being spotted onto NGM plates. Plates were left to dry overnight at room temperature before L4 animals were plated and then dissected 24 h later.

### Immunostaining and images acquisition

About 20–24 h post-L4 stage animals were dissected in 15 µl of 1xPBS and fixed with an equal amount of 2% PFA (diluted in 1xPBS from a 16% stock) for 5 min at room temperature. A 24 × 24 coverslip was gently applied and slides were submerged in liquid nitrogen for freeze-crack. Samples were placed in methanol at −20°C for 5 min and then washed thrice for 5 min at room temperature in 1xPBS with 0.1% Tween.

Blocking was performed by leaving the slides for 1 h at room temperature in 1% BSA (dissolved in 1xPBS with 0.1% Tween), followed by primary antibody treatment overnight at 4°C in a humid chamber. The following day, slides were washed in 1xPBS with 0.1% Tween thrice for 10 min each and secondary antibodies were left in incubation on the slides for 2 h at room temperature in the dark.

After 3 washes in the dark at room temperature for 10 min each, a 60 µl drop of DAPI (2 µg/ml) was placed onto the samples, allowed to stain for 1 min in the dark, and then washed for at least 20 min in 1xPBS with 0.1% Tween. A drop of 12 µl of Vectashield was placed onto the samples and a 22 × 22 coverslip was then sealed using nail polish.

The primary antibodies used in this study were: rabbit anti-pHTP-3^S285^*(*this study, absorbed against *htp-3(S285A)* mutant, 1:500), guinea pig anti-HTP-3 (Kim lab, 1:750), rabbit anti-SYP-1 (Janisiw *et al.* 2020, 1:1,500), rabbit anti-RAD-51 (this study, 1:3,000), and mouse anti His-tag (Sigma, 1:3,000).

Images were acquired with an upright fluorescence microscope Zeiss AxioImager.Z2 equipped with a Hamamatsu ORCA Flash 4.0, sCMOS sensor camera, using UPlanSApo 100×/1.4 Oil objective with Z-stacks at 0.24 µm thickness. Images were deconvolved with ZEN 3.0 Blue software (Zeiss), using “constrained iterative” algorithm at maximum strength.

### Generation of anti-phospho-HTP-3^S285^ and anti-RAD-51 antibodies

Phospho-specific antibodies against S285 in HTP-3 were produced by immunizing rabbits with the synthetic peptide CNPELDEIYFpSPGR (Genscript) in which the cysteine was added for conjugation to KLH (keyhole limpet hemocyanin). Polyclonal phosphoHTP-3^S285^ antibodies were purified and then further absorbed against *htp-3(S285A)* mutant worms to reduce background. The specificity of the antibody was assessed by immunofluorescence where lack of staining was observed in *htp-3(S285A)* mutants but not in WT animals.

The synthetic peptide CSAQASRQKKSDQEQRAADQA corresponding to the amino acids 40–59 of *C. elegans* RAD-51 isoform a (including the underlined cysteine required for conjugation to KLH) was used to perform 4 immunization rounds in 2 rabbits (Genscript). RAD-51 polyclonal antibodies were separately affinity purified from raw sera of both animals against the same synthetic peptide employed as the immunogen. The specificity of the antibody was assessed by immunostaining of *spo-11* mutants in which, unlike WT control animals, detection of RAD-51 foci was abrogated due to the absence of physiological DSBs.

### Immunoprecipitation and sample preparation for mass spectrometry

Nuclear extracts from *parg-1::GFP* and untagged WT animals were produced as detailed in Silva *et al.* (2014). One milligram of pooled nuclear-soluble and chromatin-bound fractions were used for GFP immunoprecipitations by employing agarose GFP-traps (Chromotek), which had been pre-equilibrated in Buffer D [20% glycerol, 0.2 mM EDTA pH 8, 150 mM KCl, 20 mM Hepes-KOH (pH 7.9), and 0.2% Triton X-100, supplemented with protease inhibitor cocktail (Roche)]. Incubation of beads with the extracts was carried out over night at 4°C on a rotating shaker. The following day, the beads were spun down and washed extensively in Buffer D (without Triton X-100) before an equal amount of 2× Laemmli buffer was added. Samples were boiled for 10 min and then the immunoprecipitated complexes were separated on a 4–12% acrylamide gel.

For mass spectrometry analysis, agarose beads were resuspended in 30 µl elution buffer (2 M urea, 50 mM ammonium bicarbonate), disulfide bonds reduced with 10 mM dithiothreitol for 30 min at room temperature, and then alkylated with 25 mM iodoacetamide for 15 min in the dark. After quenching with another 5 mM dithiothreitol, 150 ng of trypsin (Trypsin Gold, Promega) was added followed by 90-min incubation at room temperature in the dark. The supernatant without beads was transferred to a new tube and another 30 µl of elution buffer was added to the beads. The supernatants without beads were combined, diluted to 1 M urea concentration, and another 150 ng of trypsin was added before incubation at 37°C in the dark overnight. The digest was stopped by the addition of trifluoroacetic acid to a final concentration of 1%, and the peptides were desalted using C18 Stagetips (Rappsilber *et al.* 2007).

Peptides were separated on an Ultimate 3000 RSLC nano-flow chromatography system (Thermo-Fisher), using a precolumn for sample loading (Acclaim PepMap C18, 2 cm × 0.1 mm, 5 μm, Thermo-Fisher), and a C18 analytical column (Acclaim PepMap C18, 50 cm × 0.75 mm, 2 μm, Thermo-Fisher), applying a segmented linear gradient from 2% to 35% and finally 80% solvent B (80% acetonitrile, 0.1% formic acid; solvent A 0.1% formic acid) at a flow rate of 230 nl/min over 120 min. Eluting peptides were analyzed on a Q Exactive HF Orbitrap mass spectrometer (Thermo Fisher), which was coupled to the column with a nano-spray ion-source using coated emitter tips (New Objective).

### Mass spectrometry data acquisition and analysis

The mass spectrometer was operated in data-dependent acquisition mode, survey scans were obtained in a mass range of 380–1,650 m/z with lock mass activated, at a resolution of 120k at 200 m/z and an AGC target value of 3E6. The 10 most intense ions were selected with an isolation width of 2 m/z, fragmented in the HCD cell at 27% collision energy and the spectra recorded for max. 250 ms at a target value of 1E5 and a resolution of 30k. Peptides with a charge of +1 or >+6 were excluded from fragmentation, the peptide match feature was set to preferred, the exclude isotopes feature enabled, and selected precursors were dynamically excluded from repeated sampling for 30 s.

Raw data were processed using the MaxQuant software package **(**version 1.5.5.1; Tyanova *et al.* 2016) and the Uniprot *C. elegans* reference proteome (www.uniprot.org), as well as a database of most common lab contaminants. The search was performed with full trypsin specificity and a maximum of 2 missed cleavages at a protein and peptide spectrum match false discovery rate of 1%. Carbamidomethylation of cysteine residues was set as fixed, phosphorylation (serine, threonine, or tyrosine), oxidation (methionine), and N-terminal acetylation as variable modifications. Label-free quantification and the “match between runs” feature were activated—all other parameters were left at default. The spectrum supporting phosphorylation of pHTP-3^S285^ was validated manually.

### Protein expression and in vitro kinase assay

Recombinant HTP-3 protein was generated by cloning full-length cDNAs of *htp-3* into pTrcHis Topo (Invitrogen, catalog no. K4410-01) bacterial expression vectors to generate N-terminally 6× His-tagged proteins (Das *et al.* 2020). Mutant HTP-3^S285A^ protein was generated through site-directed mutagenesis as described (Arur *et al.* 2009). Positive clones were then sequence verified. Recombinant proteins were expressed in BL21(DE3) (Sigma-Aldrich) cells at 37°C by using 1-mM isopropyl β-d-1-thiogalactopyranoside (dioxane free) for 3 h. Proteins were then purified by using Ni–nitrilotriacetic acid resin (Thermo scientific, no. 88221). The expression of proteins was confirmed by Western blot analysis with anti-His (Sigma-Aldrich, no. H1029). In vitro kinase assay was performed using purified ERK2 kinase (New England Biolabs, catalog no. P6080S), and the purified recombinant proteins according to the methods previously described (Arur *et al.* 2009). After phosphotransfer, the proteins were resolved onto a 10% SDS–polyacrylamide gel electrophoresis (Bio-Rad, catalog no. 4561033). The gel was then dried at 60°C under vacuum for 1 h and exposed to the autoradiographic film (Sigma-Aldrich, catalog no. 864 6770) for 4 h at −80°C followed by the development of the film using the Kodak X-OMAT 2000A processor machine.

## Results and discussion

### HORMA-domain-containing protein HTP-3 is phosphorylated at S285 in vivo

We have recently shown that in *C. elegans*, the PARG-1/PARG protein, ortholog to mammalian poly(ADP-ribose)glycohydrolase, localizes along both the central and lateral elements of the SC, and its function is important for the induction of optimal levels of SPO-11-dependent DSBs and to promote their repair through HR (Janisiw *et al.* 2020). Furthermore, PARG-1*::*GFP requires HTP-3 to be properly loaded onto the chromosomes and coimmunoprecipitates with factors localizing along the SC (Janisiw *et al.* 2020).

**Fig. 1.**
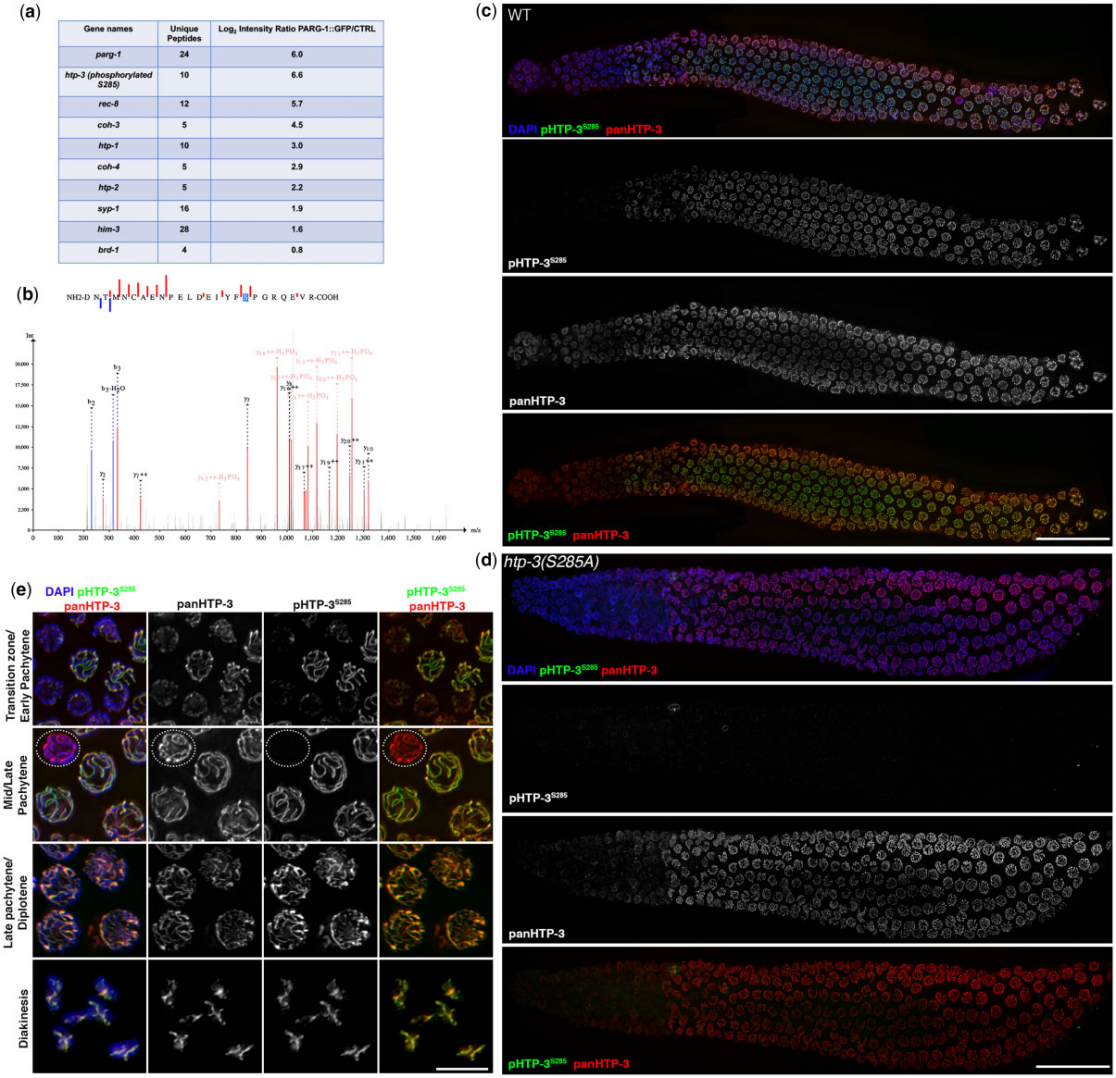
Phospho-HTP-3^S285^ accumulation follows total HTP-3 staining pattern. a) Table showing several of the putative interactors identified by mass spectrometry analysis on the PARG-1::GFP pull downs. b) Annotated fragmentation mass spectrum of the HTP-3 peptide carrying pS285. Red and blue lines indicate b- and y-ions, respectively, y ions with phosphate losses are marked in light red. The illustration was generated using PeptideShaker ver. 2.2.0 (Vaudel *et al.* 2015). c) Whole-mount gonad stained with panHTP-3 and phosphoHTP-3^S285^ antibodies. Scale bar 20 µm. d) *htp-3(S285A)* mutants stained with panHTP-3 and phosphoHTP-3^S285^ antibodies showing specificity of the phospho-antibody. Scale bar 20 µm. e) Insets showing nuclei at different stages of meiotic prophase I. Note that pHTP-3^S285^ antibody staining is absent in apoptotic cells (dotted circles). Scale bar 5 µm.

In an effort to identify novel interactors of PARG-1 *in vivo*, we performed GFP-immunoprecipitation on PARG-1*::*GFP animals followed by mass spectrometry analysis (Janisiw *et al.* 2020). We observed that PARG-1*::*GFP coimmunoprecipitated with a phosphorylated form of HTP-3 at Serine 285 (Fig. 1, a and b).

To determine whether HTP-3 is phosphorylated at S285 *in vivo*, we generated a specific phospho-HTP-3^S285^ antibody and performed immunofluorescence analysis.

Phospho-specific HTP-3^S285^ was localized to nuclei in meiotic prophase I (Fig. 1c) and the signal was abolished in the *htp-3(S285A)* mutant (Fig. 1d, Supplementary Fig. 1), confirming that the antibody is specific to the phosphorylated form of HTP-3^S285^.

**Fig. 2.**
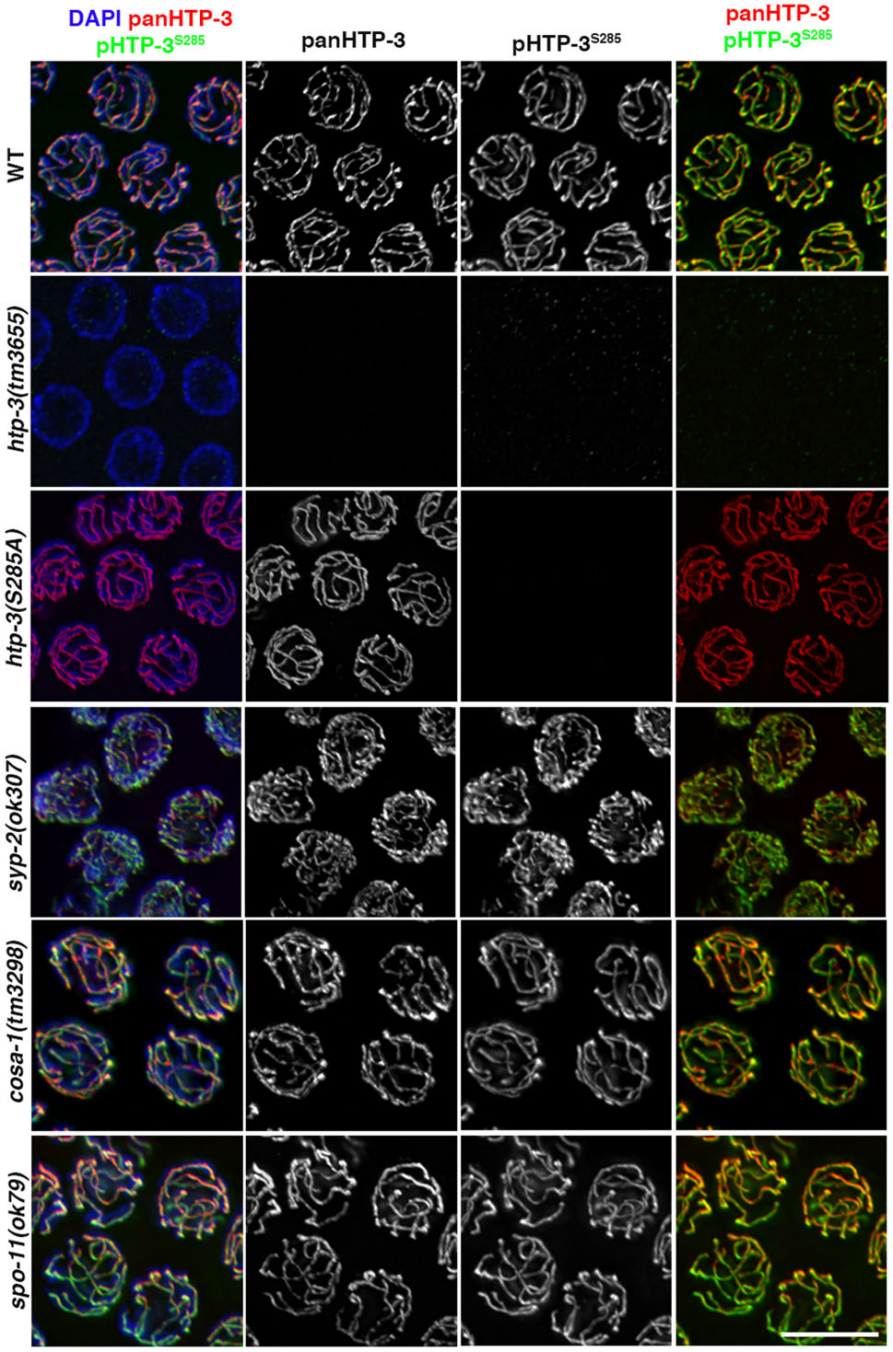
Phosphorylation of HTP-3^S285^ is independent of synapsis and recombination. Mid-pachytene nuclei stained with pHTP-3^S285^ and panHTP-3 antibodies in the indicated mutant backgrounds. Scale bar 5 µm.

The expression of phospho-HTP-3^S285^ overlaps with the pan-HTP-3 expression across the distal-proximal axis of the gonad (Fig. 1, c and e). Careful examination of the two localization patterns revealed that at meiosis onset the pHTP-3^S285^ staining was rather uneven, relative to total HTP-3; most nuclei displayed a patchy localization of pHTP-3^S285^, with only a subset of them showing the phospho-staining along the whole length of the chromosome (Fig. 1e, transition zone/Early pachytene). Chromosome axes are established upstream to synapsis and therefore lateral elements such as HTP-3 are loaded earlier than the SYP proteins (Colaiácovo *et al.* 2003; Couteau *et al.* 2004; Martinez-Perez and Villeneuve 2005; Goodyer *et al.* 2008). At this stage, the establishment of the SC occurs gradually (Colaiácovo *et al.* 2003), thus, we hypothesize that phosphorylation of HTP-3^S285^ might rely upon the completion of synapsis, which would be consistent with the gradual increase in its loading along the chromosomes (Fig. 2). By the early pachytene stage, the pHTP-3^S285^ staining was no longer distinguishable from the panHTP-3 expression except in the cells undergoing physiological apoptotic cell death, where pHTP-3^S285^ was absent (Fig. 1e, mid/late-pachytene, dotted circle). Thus, we conclude that HTP-3 is phosphorylated at S285 in vivo during meiotic prophase I.

### Phosphorylation of HTP-3^S285^ occurs independently of synapsis, DSBs, and CO establishment

Assembly of the SC central elements depends on upstream axes morphogenesis and cohesins (Pasierbek 2001; Couteau *et al.* 2004; Goodyer *et al.* 2008; Severson and Meyer 2014), and it ensues from loading interdependency amongst its components (SYP-1/6). Thus, the removal of a single member of this protein family blocks the establishment of synapsis (MacQueen *et al.*, 2002; Colaiácovo *et al.*, 2003; Smolikov *et al.*, 2009, 2007; Hurlock *et al.*, 2020; Zhang *et al.*, 2020).

Given the gradual recruitment of p-HTP-3^S285^ in the nuclei at meiosis entry, we wondered whether HTP-3^S285^ phosphorylation was dependent on early events that occur during meiotic progression, such as the establishment of synapsis and formation of physiological DSBs, respectively. To assess whether phosphorylation of HTP-3 at Ser285 required the establishment of synapsis, we performed immunostaining analysis of pHTP-3^S285^ in *syp-2* mutants. We observed that pHTP-3^S285^ was detectable along chromosomes upon loss of *syp-2* indicating that its localization is independent of SC establishment (Fig. 2).

In *C. elegans*, SC formation takes place independently of induction of SPO-11-mediated DSBs (Dernburg *et al.* 1998). Programmed DSB induction relies on the catalytic activity of the topoisomerase-like SPO-11, which exerts its function in combination with several cofactors in worms (Reddy and Villeneuve 2004; Wagner *et al.* 2010; Meneely *et al.* 2012; Rosu *et al.* 2013; Stamper *et al.* 2013; Hinman *et al.* 2021). Importantly, deletion of *htp-3* impairs meiotic break formation, most likely due to its activity in recruiting the MRN/X complex or perhaps a direct role in promoting loading of SPO-11 itself (Goodyer *et al.* 2008). Previous studies analyzed RAD-51 foci and indirectly posited that induction of DSBs takes place at early meiotic entry (Alpi *et al.* 2003; Colaiácovo *et al.* 2003). To determine whether HTP-3 phosphorylation is triggered by CO formation, we analyzed the localization of pHTP-3^S285^ in *spo-11* mutants. However, removal of *spo-11* did not affect pHTP-3^S285^ localization, suggesting that phosphorylation and localization of HTP-3 are independent of physiological DNA damage (Fig. 2).

The cyclin homolog COSA-1/CNTD1 is essential to convert recombination intermediates into mature COs, and its depletion leads to nearly complete lack of chiasmata in both worms and mice (Yokoo *et al.* 2012; Holloway *et al.* 2014). To determine whether HTP-3 phosphorylation is triggered by CO formation, we analyzed its localization in the *cosa-1* mutants. We did not observe any obvious aberrations in pHTP-3^285^ localization pattern in the *cosa-1* mutants (Fig. 2).

Taken together, these results indicate that phosphorylation of HTP-3 at Ser285 is independent of synapsis and COs, and it does not require SPO-11-mediated DSBs, suggesting that this occurs as an early event at meiosis onset, which most likely takes place either contemporaneously or immediately after axes morphogenesis.

### Phosphorylation of HTP-3^S285^ is highly redundant

**Fig. 3.**
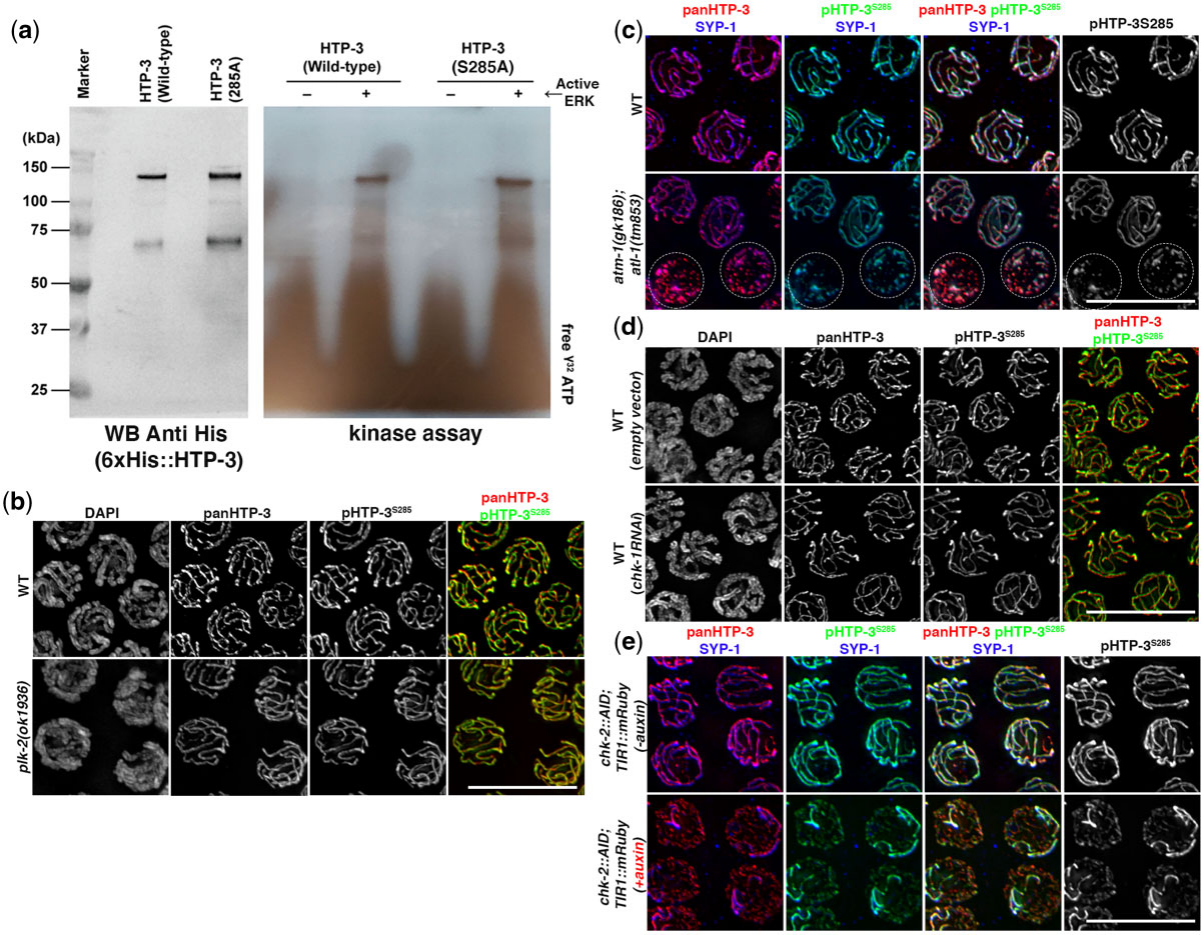
HTP-3^S285^ phosphorylation is independent of ERK, *plk-2*, *chk-1*/*chk-2*, and the DNA damage kinases *atm-1* and *atl-1*. a) *In vitro* kinase assay of HTP-3 wild-type and HTP-3^S285A^ mutants with active recombinant ERK2. b) Mid-pachytene nuclei stained for pHTP-3^S285^ and panHTP-3 in *plk-2(ok1936)*. Scale bar 10 µm. c) HTP-3^S285^ phosphorylation in ATM and ATR mutants. Dotted circles indicate nuclei with impaired axes morphogenesis. Scale bar 10 µm. d) HTP-3^S285^ phosphorylation in *chk-1^(RNAi)^*. Scale bar 10 µm. e) HTP-3^S285^ phosphorylation upon auxin-induced degradation of CHK-2. Scale bar 10 µm.

Having confirmed the phosphorylation of HTP-3 at Ser285 *in vivo*, we then wanted to identify the kinase/s driving this modification. ERK/MPK-1 phosphorylates HTP-3 *in vitro* (Das *et al.* 2020), however, the site has not yet been determined. Therefore, we investigated whether HTP-3^S285^ is phosphorylated by ERK/MPK-1. To test this, we performed an *in vitro* kinase assay using bacterially expressed HTP-3^WT^ and HTP-3^S285A^ proteins, employing active ERK2 enzyme (Fig. 3). We observed that the HTP-3 Ser285Ala mutant protein is phosphorylated by active ERK *in vitro* (Fig. 3a), suggesting that S285 is a phosphor-acceptor for ERK2.

Next, we performed pHTP-3^S285^ staining in kinase mutants known to exert roles during meiotic progression in worms. Specifically, we assayed for the involvement of *plk-2*, which functions partially redundantly with *plk-1* and is known to regulate pairing and synapsis (Harper *et al.* 2011; Labella *et al.* 2011); *chk-1* and *chk-2* mediate the DNA damage response and while *chk-1* functions more prominently during mitosis, *chk-2* promotes several key meiotic processes in worms, including induction of DSBs and licensing of the SC (MacQueen and Villeneuve 2001; Martinez-Perez and Villeneuve 2005; Kim *et al.* 2015). Finally, *atm-1/ATM* and *atl-1/ATR* function in a partially redundant manner to regulate DNA damage response: *atl-1* is critical for mitotic replication and null mutants display complete sterility, while *atm-1* mutants are viable, suggesting that the ATM-1 function is not essential during germ cell development (Garcia-Muse and Boulton 2005). Overall, we did not observe loss of pHTP-3^285^ phosphorylation in *plk-2(ok1936)* mutants (Fig. 3b), *atm-1(gk186); atl-1(tm853)* double mutants (Fig. 3c), upon RNAi-mediated depletion of *chk-1* (Fig. 3d, Supplementary Fig. 2), and auxin-induced degradation of CHK-2 using the *chk-2::AID; TIR1::mCherry* line (Castellano-Pozo *et al.* 2020; Fig. 3e). Together, these data suggest that pHTP-3^285^ is not phosphorylated by any of these kinases independently.

**Fig. 4.**
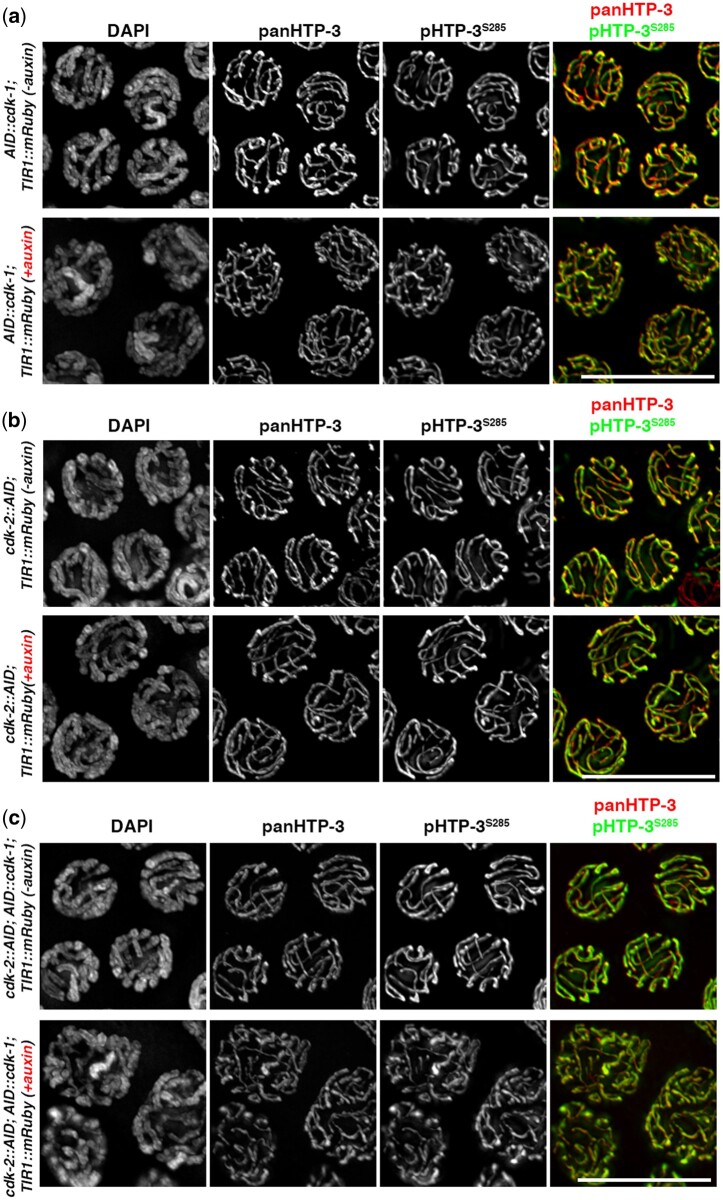
Phosphorylation of HTP-3^S285^ does not require *cdk-1* and *cdk-2*. HTP-3^S285^ phosphorylation in mutant germlines upon loss of *cdk-1* (a), *cdk-2* (b), or both (c). Scale bar 10 µm.

Recent work has shown that the 2 cyclin-dependent kinases CDK-1/2 perform crucial functions in the *C. elegans* germline, by promoting phosphorylation of the SC component SYP-1 and by exerting a direct regulatory function on pro-CO factors, thus promoting chiasmata formation (Brandt *et al.* 2020; Haversat *et al.* 2021; Zhang *et al.* 2021). We tested whether CDK1/2 mediates pHTP-3^285^ phosphorylation using a *cdk-1/2*—AID-tagged lines, in which degradation of CDK-1/2 was elicited by exposure to auxin. We observed that depletion of either *cdk-1* (Fig. 4a) or *cdk-2* (Fig. 4b), as well as contemporaneous removal of both kinases (Fig. 4c) did not affect the phosphorylation and localization of HTP-3^S285^ suggesting that S285 is likely not a substrate of these 2 kinases.

Altogether, these results indicate that none of the major kinases that have been shown to exert important roles during meiotic prophase I in the *C. elegans* gonad regulate phosphorylation of HTP-3^S285^, suggesting that this residue may be recognized by a different kinase/s or subjected to a highly redundant phospho-regulation.

### Phosphorylation of HTP-3^S285^ is dispensable for chiasmata formation

**Fig. 5.**
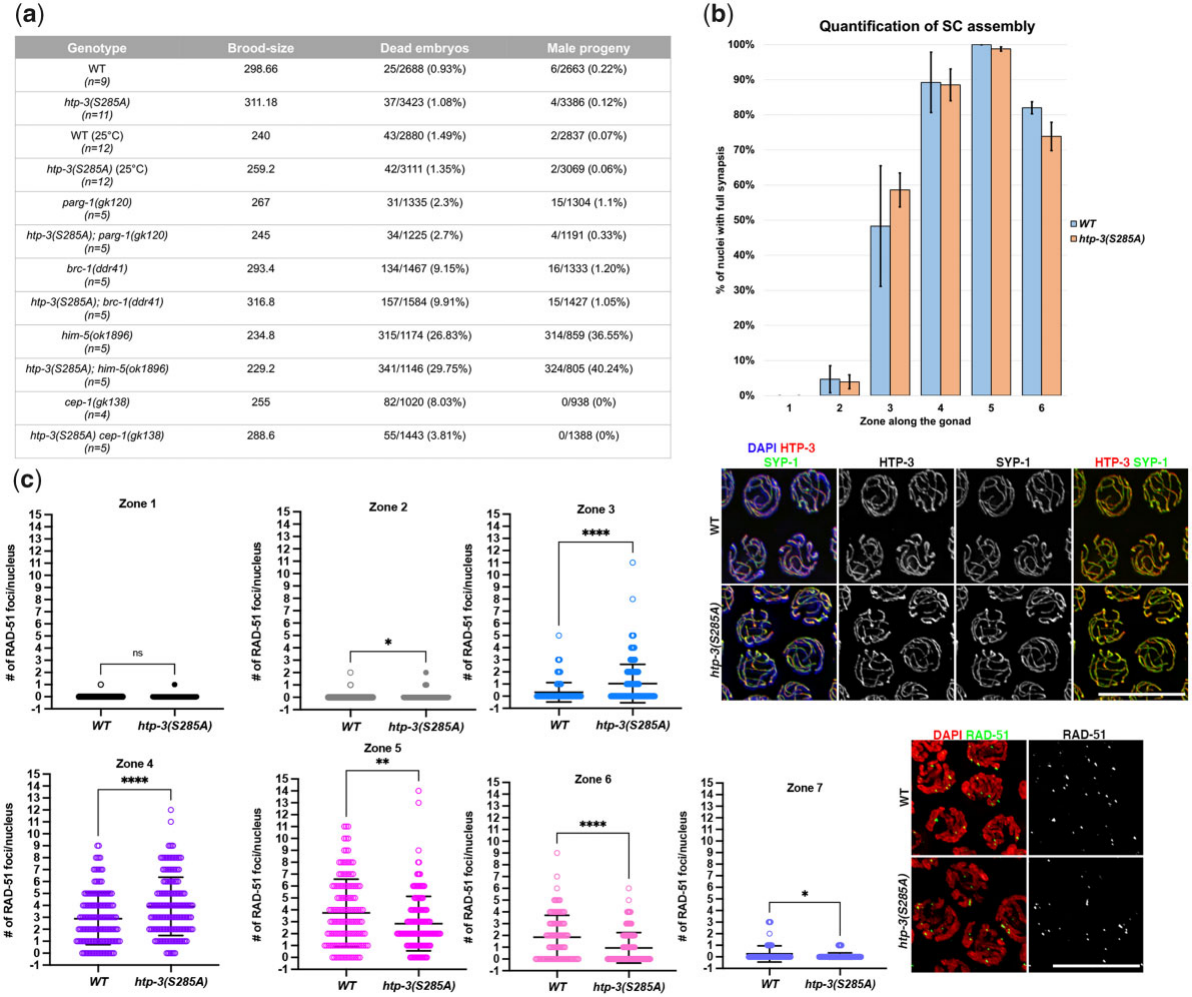
*htp-3(S285A)* mutants display normal levels of fertility. a) Analysis of viability, brood size, and male progeny in the indicated mutants. *n* indicates the number of worms analyzed. b) Top: quantification of synapsis in the *htp-3(S285A)* mutants. Bottom: representative examples of mid-pachytene nuclei from the indicated genotypes labeled with HTP-3 and SYP-1. Scale bar 10 µm. c) Time-course analysis of RAD-51 foci formation and removal in *htp-3(S285A)* mutants and wild-type controls. Gonads were divided into 7 equal regions from the mitotic tip to diplotene entry and the number of RAD-51 foci in each nucleus was counted. Bars in the charts indicate mean and standard deviation (*ns*, nonsignificant, ****P < 0.0001, **P = 0.0072, *P = 0.047 as calculated by *T*-test). Insets show representative images of early-pachytene nuclei stained with anti-RAD-51 antibodies in the indicated genotypes. Scale bar 10 µm.

To determine the biological significance of HTP-3^S285^ phosphorylation, we generated an unphosphorylatable HTP-3 by changing the Serine 285 to Alanine (Supplementary Fig. 3) using CRISPR/Cas9 method. We assessed the hatching rates in the *htp-3(S285A)* unphosphorylatable mutant worms, as well as monitored the establishment of synapsis and induction/resolution of the recombination intermediates by analyzing RAD-51 dynamics. We found that compared to wild type, there are no defects in either embryonic viability or generation of male progeny (Him phenotype) in the *htp-3(S285A)* mutant (Fig. 5a), indicating that HTP-3 phosphorylation at this site is not essential to preserve fertility. We also performed viability analysis in worms grown at 25°C, known to induce destabilization of the SC and increased lethality in DNA repair-defective mutants. However, we did not observe any significant differences between WT and *htp-3(S285A)* mutants (Fig. 5a).

Because removal of pHTP-3^S285^ did not cause obvious defects, we wondered whether the phospho-mutant may be sensitive to the lack of factors involved in DSB induction/repair, intersister DSB repair and DNA damage-induced apoptosis. To test this, we generated the *htp-3(S285A); parg-1(gk120)* and the *htp-3(S285A); him-5(ok1896), htp-3(S285A); brc-1(ddr41)* and *htp-3(S285A) cep-1(gk138)* double-mutant backgrounds, respectively (Schumacher *et al.* 2001; Boulton *et al.* 2004; Adamo *et al.* 2008; Meneely *et al.* 2012; Janisiw *et al.* 2018, 2020). Lack of *parg-1* weakens DSB formation, as its removal from genetic backgrounds bearing suboptimal levels of meiotic breaks exacerbates CO defects. *him-5* is important to achieve abundant DSBs and abrogation of its function dramatically impairs the establishment of CO on the chromosome X. Embryonic lethality and male progeny in the *htp-3(S285A); parg-1* and *htp-3(S285A); him-5* double mutants were comparable to the relative single mutant controls (Fig. 5a), suggesting no additive or synthetic interaction between HTP-3 phosphorylation and the meiotic pathways tested.

*Caenorhabditis* *elegans brc-1*, homolog to mammalian BRCA1, is essential for DSB repair through intersister recombination, and *cep-1*, homolog to mammalian *p53*, is involved in DNA damage-induced germ cell death in *C. elegans*. While single mutants in either *brc-1* or *cep-1* are viable, double mutants between either of these and genes that affect chiasma formation (e.g. *him-14*/*msh-4*) or DSB induction (e.g. *him-5*) lead to synthetic mutant phenotypes and defects in the resulting progeny (Adamo *et al.* 2008). Thus, we tested whether there is any synthetic lethal interaction between *htp-3(S285A)* and *brc-1* or *cep-1*. We observed that the *htp-3(S285A); brc-1* and *htp-3(S285A) cep-1* doubles did not differ from the *brc-1* and *cep-1* single mutants, suggesting that neither intersister-mediated DSB repair nor DNA damage-dependent apoptosis exerts essential functions in *htp-3(S285A)* phospho-mutant (Fig. 5a).

Additionally, we did not observe any defects in the establishment of synapsis (Fig. 5b) or in the formation of recombination intermediates (Fig. 5c), as indicated by colocalization between panHTP-3 and SYP-1 and comparable number of RAD-51 foci in the *htp-3(S285A)* mutants as in WT controls. However, we found a small, although statistically significant acceleration in the RAD-51 foci turnover. The RAD-51 foci peaked and disappeared earlier in the *htp-3(S285A)* mutants compared to the WT animals (Fig. 5c, zones 3–6). This could indicate a role for HTP-3^S285^ phosphorylation in regulating the timing of DSB induction or alternatively in influencing the DSB repair kinetics.

Altogether, these data demonstrate that HTP-3 is phosphorylated at Ser285 during meiotic prophase I. It is interesting to note that HTP-3 S285 is not conserved in the *Caenorhabditis* species, suggesting that its regulation may be specific to *C. elegans*. Functionally, HTP-3 phosphorylation at Ser285 is dispensable for the execution of the HTP-3-dependent functions during axes morphogenesis and DSB induction. We believe that phospho-regulation of HTP-3 might proceed in a highly redundant fashion, since lack of different kinases did not abrogate phosphorylation at Ser285 and further, the functional requirements imposed by HTP-3 phosphorylation could be shared through multiple accessory sites yet to be identified.

## Data availability

The data underlying this article are available in the article and in its online supplementary material. All reagents are available upon request.

Supplemental material is available at *G3* online.

## Supplementary Material

jkac079_Supplemental_Material

## References

[jkac079-B1] Adamo A , Montemauri P, Silva N, Ward JD, Boulton SJ, La Volpe A. BRC‐1 acts in the inter‐sister pathway of meiotic double‐strand break repair. EMBO Rep. 2008;9(3):287–292. 18219312 10.1038/sj.embor.7401167 PMC2267377

[jkac079-B2] Alpi A , Pasierbek P, Gartner A, Loidl J. Genetic and cytological characterization of the recombination protein RAD-51 in *Caenorhabditis elegans*. Chromosoma. 2003;112(1):6–16. 12684824 10.1007/s00412-003-0237-5

[jkac079-B3] Arur S , Ohmachi M, Nayak S, Hayes M, Miranda A, Hay A, Golden A, Schedl T. Multiple ERK substrates execute single biological processes in *Caenorhabditis elegans* germ-line development. Proc Natl Acad Sci U S A. 2009;106(12):4776–4781. 19264959 10.1073/pnas.0812285106 PMC2660749

[jkac079-B4] Boulton SJ , Martin JS, Polanowska J, Hill DE, Gartner A, Vidal M. BRCA1/BARD1 orthologs required for DNA repair in *Caenorhabditis elegans*. Curr Biol. 2004;14(1):33–39. 14711411 10.1016/j.cub.2003.11.029

[jkac079-B5] Brandt JN , Hussey KA, Kim Y. Spatial and temporal control of targeting Polo-like kinase during meiotic prophase. J Cell Biol. 2020;219(11):e202006094. 32997737 10.1083/jcb.202006094 PMC7594494

[jkac079-B6] Brenner S. The genetics of *Caenorhabditis elegans*. Genetics. 1974;77(1):71–94. 4366476 10.1093/genetics/77.1.71 PMC1213120

[jkac079-B7] Castellano-Pozo M , Pacheco S, Sioutas G, Jaso-Tamame AL, Dore MH, Karimi MM, Martinez-Perez E. Surveillance of cohesin-supported chromosome structure controls meiotic progression. Nat Commun. 2020;11(1):4345. 32859945 10.1038/s41467-020-18219-9 PMC7455720

[jkac079-B8] Colaiácovo MP , MacQueen AJ, Martinez-Perez E, McDonald K, Adamo A, La Volpe A, Villeneuve AM. Synaptonemal complex assembly in *C. elegans* is dispensable for loading strand-exchange proteins but critical for proper completion of recombination. Dev Cell. 2003;5(3):463–474. 12967565 10.1016/s1534-5807(03)00232-6

[jkac079-B9] Couteau F , Nabeshima K, Villeneuve A, Zetka M. A component of *C. elegans* meiotic chromosome axes at the interface of homolog alignment, synapsis, nuclear reorganization, and recombination. Curr Biol. 2004;14(7):585–592. 15062099 10.1016/j.cub.2004.03.033

[jkac079-B10] Das D , Chen S-Y, Arur S. ERK phosphorylates chromosomal axis component HORMA domain protein HTP-1 to regulate oocyte numbers. Sci Adv. 2020;6(44):eabc5580. 33127680 10.1126/sciadv.abc5580 PMC7608811

[jkac079-B11] Dernburg AF , McDonald K, Moulder G, Barstead R, Dresser M, Villeneuve AM. Meiotic recombination in *C. elegans* initiates by a conserved mechanism and is dispensable for homologous chromosome synapsis. Cell. 1998;94(3):387–398. 9708740 10.1016/s0092-8674(00)81481-6

[jkac079-B12] Garcia-Muse T , Boulton SJ. Distinct modes of ATR activation after replication stress and DNA double-strand breaks in *Caenorhabditis elegans*. EMBO J. 2005;24(24):4345–4355. 16319925 10.1038/sj.emboj.7600896 PMC1356337

[jkac079-B13] Goodyer W , Kaitna S, Couteau F, Ward JD, Boulton SJ, Zetka M. HTP-3 links DSB formation with homolog pairing and crossing over during *C. elegans* meiosis. Dev Cell. 2008;14(2):263–274. 18267094 10.1016/j.devcel.2007.11.016

[jkac079-B14] Harper NC , Rillo R, Jover-Gil S, Assaf ZJ, Bhalla N, Dernburg AF. Pairing centers recruit a polo-like kinase to orchestrate meiotic chromosome dynamics in *C. elegans*. Dev Cell. 2011;21(5):934–947. 22018922 10.1016/j.devcel.2011.09.001 PMC4343031

[jkac079-B15] Haversat J , Woglar A, Klatt K, Akerib CC, Roberts V, Salazar CC, Chen SY, Arur S, Villeneuve AM, Kim Y. Robust designation of meiotic crossover sites by CDK-2 through phosphorylation of the MutSγ complex. https://doi.org/10.1101/2021.08.31.458431 10.1073/pnas.2117865119 PMC9173770 35576467

[jkac079-B16] Hinman AW , Yeh H-Y, Roelens B, Yamaya K, Woglar A, Bourbon H-M G, Chi P, Villeneuve AM. *Caenorhabditis elegans* DSB-3 reveals conservation and divergence among protein complexes promoting meiotic double-strand breaks. Proc Natl Acad Sci USA. 2021;118:e2109306118. 34389685 10.1073/pnas.2109306118 PMC8379965

[jkac079-B17] Holloway JK , Sun X, Yokoo R, Villeneuve AM, Cohen PE. Mammalian CNTD1 is critical for meiotic crossover maturation and deselection of excess precrossover sites. J Cell Biol. 2014;205(5):633–641. 24891606 10.1083/jcb.201401122 PMC4050721

[jkac079-B18] Hurlock ME , Čavka I, Kursel LE, Haversat J, Wooten M, Nizami Z, Turniansky R, Hoess P, Ries J, Gall JG, et al Identification of novel synaptonemal complex components in *C. elegans*. J Cell Biol. 2020;219(5):e201910043. 32211899 10.1083/jcb.201910043 PMC7199856

[jkac079-B19] Janisiw E , Dello Stritto MR, Jantsch V, Silva N. BRCA1-BARD1 associate with the synaptonemal complex and pro-crossover factors and influence RAD-51 dynamics during *Caenorhabditis elegans* meiosis. PLoS Genet. 2018;14(11):e1007653. 30383754 10.1371/journal.pgen.1007653 PMC6211622

[jkac079-B20] Janisiw E , Raices M, Balmir F, Paulin LF, Baudrimont A, von Haeseler A, Yanowitz JL, Jantsch V, Silva N. Poly(ADP-ribose) glycohydrolase coordinates meiotic DNA double-strand break induction and repair independent of its catalytic activity. Nat Commun. 2020;11(1):4869. 32978394 10.1038/s41467-020-18693-1 PMC7519143

[jkac079-B21] Keeney S , Giroux CN, Kleckner N. Meiosis-specific DNA double-strand breaks are catalyzed by Spo11, a member of a widely conserved protein family. Cell. 1997;88(3):375–384. 9039264 10.1016/s0092-8674(00)81876-0

[jkac079-B22] Kim Y , Kostow N, Dernburg AF. The chromosome axis mediates feedback control of CHK-2 to ensure crossover formation in *C. elegans*. Dev Cell. 2015;35(2):247–261. 26506311 10.1016/j.devcel.2015.09.021 PMC4624198

[jkac079-B23] Labella S , Woglar A, Jantsch V, Zetka M. Polo kinases establish links between meiotic chromosomes and cytoskeletal forces essential for homolog pairing. Dev Cell. 2011;21(5):948–958. 22018921 10.1016/j.devcel.2011.07.011

[jkac079-B24] MacQueen AJ , Colaiácovo MP, McDonald K, Villeneuve AM. Synapsis-dependent and -independent mechanisms stabilize homolog pairing during meiotic prophase in *C. elegans*. Genes Dev. 2002;16(18):2428–2442. 12231631 10.1101/gad.1011602 PMC187442

[jkac079-B25] MacQueen AJ , Villeneuve AM. Nuclear reorganization and homologous chromosome pairing during meiotic prophase require *C. elegans* chk-2. Genes Dev. 2001;15(13):1674–1687. 11445542 10.1101/gad.902601 PMC312723

[jkac079-B26] Martinez-Perez E , Villeneuve AM. HTP-1-dependent constraints coordinate homolog pairing and synapsis and promote chiasma formation during *C. elegans* meiosis. Genes Dev. 2005;19(22):2727–2743. 16291646 10.1101/gad.1338505 PMC1283965

[jkac079-B27] Meneely PM , McGovern OL, Heinis FI, Yanowitz JL. Crossover distribution and frequency are regulated by *him-5* in *Caenorhabditis elegans*. Genetics. 2012;190(4):1251–1266. 22267496 10.1534/genetics.111.137463 PMC3316641

[jkac079-B28] Pasierbek P , Jantsch M, Melcher M, Schleiffer A, Schweizer D, Loidl J. A *Caenorhabditis elegans* cohesion protein with functions in meiotic chromosome pairing and disjunction. Genes Dev. 2001;15(11):1349–1360. 11390355 10.1101/gad.192701 PMC312707

[jkac079-B29] Rappsilber J , Mann M, Ishihama Y. Protocol for micro-purification, enrichment, pre-fractionation and storage of peptides for proteomics using StageTips. Nat Protoc. 2007;2(8):1896–1906. 17703201 10.1038/nprot.2007.261

[jkac079-B30] Reddy KC , Villeneuve AM. *C. elegans* HIM-17 links chromatin modification and competence for initiation of meiotic recombination. Cell. 2004;118(4):439–452. 15315757 10.1016/j.cell.2004.07.026

[jkac079-B31] Rosu S , Zawadzki KA, Stamper EL, Libuda DE, Reese AL, Dernburg AF, Villeneuve AM. The *C. elegans* DSB-2 protein reveals a regulatory network that controls competence for meiotic DSB formation and promotes crossover assurance. PLoS Genet. 2013;9(8):e1003674. 23950729 10.1371/journal.pgen.1003674 PMC3738457

[jkac079-B32] Schumacher B , Hofmann K, Boulton S, Gartner A. The *C. elegans* homolog of the p53 tumor suppressor is required for DNA damage-induced apoptosis. Curr Biol. 2001;11(21):1722–1727. 11696333 10.1016/s0960-9822(01)00534-6

[jkac079-B33] Severson AF , Ling L, van Zuylen V, Meyer BJ. The axial element protein HTP-3 promotes cohesin loading and meiotic axis assembly in *C. elegans* to implement the meiotic program of chromosome segregation. Genes Dev. 2009;23(15):1763–1778. 19574299 10.1101/gad.1808809 PMC2720254

[jkac079-B34] Severson AF , Meyer BJ. Divergent kleisin subunits of cohesin specify mechanisms to tether and release meiotic chromosomes. Elife. 2014;3:e03467. 25171895 10.7554/eLife.03467 PMC4174578

[jkac079-B35] Silva N , Ferrandiz N, Barroso C, Tognetti S, Lightfoot J, Telecan O, Encheva V, Faull P, Hanni S, Furger A, et al The fidelity of synaptonemal complex assembly is regulated by a signaling mechanism that controls early meiotic progression. Dev Cell. 2014;31(4):503–511. 25455309 10.1016/j.devcel.2014.10.001

[jkac079-B36] Smolikov S , Eizinger A, Schild-Prufert K, Hurlburt A, McDonald K, Engebrecht J, Villeneuve AM, Colaiácovo MP. SYP-3 restricts synaptonemal complex assembly to bridge paired chromosome axes during meiosis in *Caenorhabditis elegans*. Genetics. 2007;176(4):2015–2025. 17565948 10.1534/genetics.107.072413 PMC1950610

[jkac079-B37] Smolikov S , Schild-Prüfert K, Colaiácovo MP. A yeast two-hybrid screen for SYP-3 interactors identifies SYP-4, a component required for synaptonemal complex assembly and chiasma formation in *Caenorhabditis elegans* meiosis. PLoS Genet. 2009;5(10):e1000669. 19798442 10.1371/journal.pgen.1000669 PMC2742731

[jkac079-B38] Stamper EL , Rodenbusch SE, Rosu S, Ahringer J, Villeneuve AM, Dernburg AF. Identification of DSB-1, a protein required for initiation of meiotic recombination in *Caenorhabditis elegans*, illuminates a crossover assurance checkpoint. PLoS Genet. 2013;9(8):e1003679. 23990794 10.1371/journal.pgen.1003679 PMC3749324

[jkac079-B39] Tyanova S , Temu T, Cox J. The MaxQuant computational platform for mass spectrometry-based shotgun proteomics. Nat Protoc. 2016;11(12):2301–2319. 27809316 10.1038/nprot.2016.136

[jkac079-B40] Vaudel M , Burkhart JM, Zahedi RP, Oveland E, Berven FS, Sickmann A, Martens L, Barsnes H. PeptideShaker enables reanalysis of MS-derived proteomics data sets. Nat Biotechnol. 2015;33(1):22–24. 25574629 10.1038/nbt.3109

[jkac079-B41] Wagner CR , Kuervers L, Baillie DL, Yanowitz JL. xnd-1 regulates the global recombination landscape in *Caenorhabditis elegans*. Nature. 2010;467(7317):839–843. 20944745 10.1038/nature09429 PMC3045774

[jkac079-B42] Yokoo R , Zawadzki KA, Nabeshima K, Drake M, Arur S, Villeneuve AM. COSA-1 reveals robust homeostasis and separable licensing and reinforcement steps governing meiotic crossovers. Cell. 2012;149(1):75–87. 22464324 10.1016/j.cell.2012.01.052 PMC3339199

[jkac079-B43] Zhang L , Stauffer W, Zwicker D, Dernburg AF. Crossover patterning through kinase-regulated condensation and coarsening of recombination nodules. Cell Bio. 2021. 10.1038/s41556-025-01688-9 40537553

[jkac079-B44] Zhang Z , Xie S, Wang R, Guo S, Zhao Q, Nie H, Liu Y, Zhang F, Chen M, Liu L, et al Multivalent weak interactions between assembly units drive synaptonemal complex formation. J Cell Biol. 2020;219(5):e201910086. 32211900 10.1083/jcb.201910086 PMC7199860

[jkac079-B45] Zickler D , Kleckner N. Meiotic chromosomes: integrating structure and function. Annu Rev Genet. 1999;33:603–754. 10690419 10.1146/annurev.genet.33.1.603

[jkac079-B46] Zickler D , Kleckner N. Recombination, pairing, and synapsis of homologs during meiosis. Cold Spring Harb Perspect Biol. 2015;7(6):a016626. 25986558 10.1101/cshperspect.a016626 PMC4448610

