# Supplementary material for: Phosphorylation of HORMA-domain protein HTP-3 at Serine 285 is dispensable for crossover formation
Source: G3 (Bethesda). 2022 Apr 7;12(5):jkac079. doi: 10.1093/g3journal/jkac079 (PMC9073698; doi:10.1093/g3journal/jkac079)
Supplement: jkac079_Supplemental_Material [file jkac079_supplemental_material.docx]

**SUPPLEMENTARY FIGURE LEGENDS**

**Figure S1. pHTP-3S285 is specific.**

Mid- pachytene magnified nuclei from the indicated genotypes, stained by panHTP-3 and pHTP-3^S285^ antibodies. Scale bar 5μm.

**Figure S2. Assessment of *chk-1^RNAi^* efficacy.**

1. Whole-mount gonad from wild type animals fed to HT115 bacteria containing the L4440 empty vector for 48h stained for panHTP-3 and pHTP-3^S285^ shows no aberrancies in nuclear progression. Scale bar 20 μm. **(B)** Whole-mount gonad from wild type animals fed to HT115 bacteria expressing dsRNA targeting *chk-1* for 48h elicits aberrant morphology, uneven nuclear size and micronuclei in the distal tip of the germ line, indicative of impaired *chk-1* function (Garcia-Muse and Boulton 2005). Scale bar 20 μm. **(C)** and **(D)** are high magnification insets of the pre-meiotic portion of the gonad to highlight different nuclei appearance triggered by *chk-1^RNAi^*. Scale bars 20 μm

**Figure S3. Chromatogram data displaying genomic sequence of the residues mutated in the *htp-3(S285A)* mutant allele.**

The green boxes indicate silent mutations included to prevent Cas9-mediated cleavage of the repair template, whereas the red boxes show the two residues mutated to change Serine 285 to Alanine (TCA to GCG respectively).
